# The emergence of drug resistance in tumours: a characteristic which may be exploited therapeutically.

**DOI:** 10.1038/bjc.1973.49

**Published:** 1973-05

**Authors:** M. H. Tattersall


*Br. J. Cancer* (1973) **27**, 406

### *Short Communications*

### THE EMERGENCE OF DRUG RESISTANCE IN TUMOURS: A CHARACTERISTIC WHICH MAY BE EXPLOITED THERAPEUTICALLY

M. H. N. TATTERSALL

*From the Department of Haematology and M.R.C. Leukaemia Unit, Royal Postgraduate Medical School, Du Cane Road, London W12*

Received 3 January 1973. Accepted 31 January 1973

UNTIL now, attempts to identify in tumour cells a selective biochemical difference which might be susceptible to selective chemical attack have not been successful. However, studies have shown that tumour cells, unlike normal cells, frequently become resistant to cytotoxic drugs, and this recognition has led to the widespread use of multiple drugs in combination in the treatment of a variety of human malignant tumours. Combination schedules are legion and most of these are based on the association of one or more anti-metabolites with one or more alkylating agents, often with steroids and cytotoxic antibiotics and plant extracts. These schedules have invariably been derived empirically and, except in a few instances, little attention has been paid to the clinical or biochemical pharmacology of the component drugs or to their possible interaction. Moreover, there has been a blind faith in the synergisms of drugs used in combination with little concern over possible antagonisms.

Goodman's group first suggested that tumour resistance to the anti-metabolite methotrexate might be exploited therapeutically (Goodman *et al.*, 1964). They had observed that tumour cells which developed resistance to this agent frequently had increased levels of the enzyme dihydrofolate reductase (Friedkin *et al.*, 1962). It was therefore suggested that these cells would be selectively sensitive on the basis of their high reductase activity to anti-metabolite compounds which were activated by this enzyme. The homofolates are activated in this way but their use in this context has been frustrated by their chemical instability. Recently, more stable reduced homofolate compounds have been synthesized and preliminary results in mice bearing leukaemia L1210 indicate that dihydrohomofolate has greater activity against a methotrexate-resistant tumour with high levels of dihydrofolate reductase than against a methotrexate-sensitive tumour with normal levels of the enzyme (Mishra and Mead, 1972). The results shown by these workers thus substantiate the principle of an enzyme-dependent regeneration of a faulty cofactor being a useful model for obtaining selective anti-tumour effects.

The investigation of acquired drug resistance in animal tumours has led to the recognition of several possible mechanisms for resistance to any particular drug. The difficulty of obtaining suitable material for study from human tumours has prevented detailed investigation. However, Steuart and Burke (1971) have recently reported that cytidine deaminase activity is increased in leukaemic cells of patients whose disease has become resistant to treatment with cytosine arabinoside. This finding, if confirmed, identifies a potentially exploitable characteristic of human leukaemic cells. In normal human tissue cytidine deaminase activity is not great and it is perhaps for this reason that 5-fluorocytosine, a drug which is activated by deamination is, in general, a non-toxic agent (Koechlin *et al.*, 1966). This drug, which was originally synthesized by Heidelberger *et al.* (1957) and discarded as it had no useful anti-tumour effects, has in the last few years been found to be an effective anti-fungal agent. It seems clear that the toxicity of 5-fluorocytosine against fungi is due to their having an active deaminase which converts 5-fluorocytosine to 5-fluorouracil. This latter agent, following its metabolism to 5-fluorodeoxyuridine monophosphate, is an inhibitor of thymidylate synthetase. Thus, it is believed that the selective toxicity of 5-fluorocytosine in fungi and not in normal human cells is a consequence of the fungi possessing an enzyme which activates the drug to a lethal compound.

5-fluorocytosine has been used widely for the treatment of fungal disease during the past few years (Fass and Perkins, 1971). Adverse reactions reported during 5-fluorocytosine treatment have been rare, although one or two patients have become severely thrombocytopenic and neutropenic while taking the drug. No satisfactory explanation of these toxicities has been suggested, nor is it apparent that these effects were seen in patients with leukaemia who had previously been treated with cytosine arabinoside for their primary disease.

The recognition that human leukaemic cells which have become resistant to cytosine arabinoside may contain increased cytidine deaminase activity (Steuart and Burke, 1971) indicates a potential therapeutic use for 5-fluorocytosine. In this context, it is reasonable to assume that the drug would be toxic only in those cells which contained high deaminase activity and that such cells would be leukaemic.

Preliminary studies in this laboratory of the cytidine kinase and deaminase activity of human leukaemic cells have shown that deaminase activity is increased in the leukaemic cells of some patients who have been treated with cytosine arabinoside. It is planned to treat patients whose leukaemic cells have high cytidine deaminase activity with 5-fluorocytosine in the hope that this drug will be selectively toxic to their leukaemic clone of cells. It has also been observed that the cytidine kinase activity may be increased in cells with high deaminase activity, and it thus remains to be shown that the high deaminase activity reported by Steuart and Burke (1971) is more than a non-specific indication of an immature cell population being selected by chemotherapy, rather than a particular mechanism of resistance.

It is widely recognized in cancer chemotherapy clinics that while tumour cells may become resistant to repeated courses of cytotoxic therapy normal marrow and gut lining cells appear to maintain their sensitivity. It is this factor which usually limits the scale of chemotherapy in patients with drug-resistant tumours, since the large doses of cytotoxic drugs which may be required to overcome resistance are not tolerated by the normal host tissues. However, this therapeutic impasse has specific chemotherapeutic possibilities in a very general sense also. This derives from the principle of therapeutic " rescue " which has been used widely during the last few years, particularly with the antifolate drug, methotrexate. The principle of this approach, as currently practised, is that an agent which damages rapidly proliferating cells, leading to cell death, may have less drastic effects in more slowly proliferating cells provided the duration of drug exposure is short (Bruce, Meeker and Valeriote, 1966). Methotrexate, by blocking the enzyme dihydrofolate reductase, deprives cells of the reduced folate co-factors which are required for DNA synthesis (Osborne, Freeman and Huennekins, 1958). Reduced folate (folinic acid) can be administered after a 24–36 hour methotrexate infusion, and this will immediately restore the folate pools in cells which have not been irreparably damaged by the folate depletion. Experience has indicated that this population of cells is composed mainly of normal and not tumour cells. Using this approach, impressive cancer therapeutic successes have been reported (Capizzi *et al.*, 1970; Djerassi *et al.*, 1972).

In the case of methotrexate-resistant tumour cells and sensitive marrow and gut cells, methotrexate therapy will inhibit DNA synthesis in the normal cells but have little effect on the resistant tumour cells. In this situation, it seems possible that resistant tumour cells will be selectively sensitive to agents which lethally damage cells undergoing DNA synthesis. Thus, prior administration of a methotrexate infusion would protect normal cells from the lethal effects of subsequently administered cytosine arabinoside, and the cytosine arabinoside would be selectively toxic to the resistant cells synthesizing DNA. Following 24–36 hours of methotrexate infusion, the normal marrow and gut cells would be " rescued " with folinic acid.

Thus, the emergence of drug resistance in tumours may be turned to therapeutic advantage. It is clearly important that the mechanisms of cytotoxic drug action and resistance in human tumour cells should be studied, not only so that drugs may be administered in rational combination schedules, but also so that possible selective advantage may be taken of the potentiality of tumour cells to develop alternate metabolic paths.

M. H. N. Tattersall is a Medical Research Council Clinical Research Fellow.

### REFERENCES

BRUCE, W. R., MEEKER, B. E. & VALERIOTE, F. A. (1966) Comparison of the Sensitivity of Normal Haematopoietic and Transplanted Lymphoma Colony-Forming Cells to Chemotherapeutic Agents Administered *in vivo*. *J. natn. Cancer Inst.*, **37**, 233.

CAPIZZI, R. L., DECONTI, R. C., MARSH, J. C. & BERTINO, J. R. (1970) Methotrexate Therapy of Head and Neck Cancer: Improvement in Therapeutic Index by the use of Leucovorin " Rescue ". *Cancer Res.*, **30**, 1782.

DJERASSI, I., ROMINGER, C. J., KIM, J. S., TURCHI, J., SUVANSRI, V. & HUGHES, D. (1972) Phase I Study of High Doses of Methotrexate with Citrovorum Factor in Patients with Lung Cancer. *Cancer, N.Y.*, **30**, 22.

FASS, R. J. & PERKINS, R. L. (1971) 5-Fluorocytosine in the Treatment of Cryptococcal and Candida Mycoses. *Ann. intern. Med.*, **74**, 535.

FRIEDKIN, M., CRAWFORD, E., HUMPHREYS, S. R. & GOLDIN, A. (1962) The Association of Increased Dihydrofolate Reductase with Amethopterin Resistance in Mouse Leukaemia. *Cancer Res.*, **22**, 600.

GOODMAN, L., DEGRAW, J., KISLIUK, R. L., FRIEDKIN, M., PASTORE, E. J., GRAWFORD, E. J., PLANTE, L. T., AL-NAHAS, A., MORNINGSTAR, J. F., KWEK, G., WILSON, L., DONOVAN, E. F. & RATZAN, J. (1964) Tetrahydrohomofolate, a Specific Inhibitor of Thymidylate Synthetase. *J. Am. chem. Soc.*, **86**, 308.

HEIDELBERGER, C., CHAUDHURI, N. K., DANNEBERG, P., MOOREN, D., GRIESBACH, L., DUSCHINSKY, R., SCHNITZER, R., PLEVEN, E. & STEINER, J. (1957) Fluorinated Pyrimidines, a New Class of Tumour Inhibitory Compounds. *Nature, Lond.*, **179**, 663.

KOECHLIN, B. A., RUBIO, F., PALMER, S., GALVIEL, T. & DUSCHINSKY, R. (1966) The Metabolism of 5 Fluorocytosine-$2^{14}C$, and of Cytosine-$^{14}C$ in the Rat, and the Disposition of 5 Fluorocytosine-$2^{14}C$ in Man. *Biochem. Pharmac.*, **15**, 435.

MISHRA, L. G. & MEAD, J. A. R. (1972) Further Evaluation of the Antitumour Activity of Homofolate and its Reduced Derivatives Against Methotrexate-Resistant Tumours. *Chemotherapy*, **17**, 283.

OSBORN, M. J., FREEMAN, M. & HUENNEKENS, F. M. (1958) Inhibition of Dihydrofolic Reductase by Aminopterin and Amethopterin. *Proc. Soc. exp. Biol. Med.*, **97**, 429.

STEUART, C. D. & BURKE, P. J. (1971) Cytidine Deaminase and the Development of Resistance to Arabinosyl Cytosine. *Nature, New Biol.*, **233**, 109.


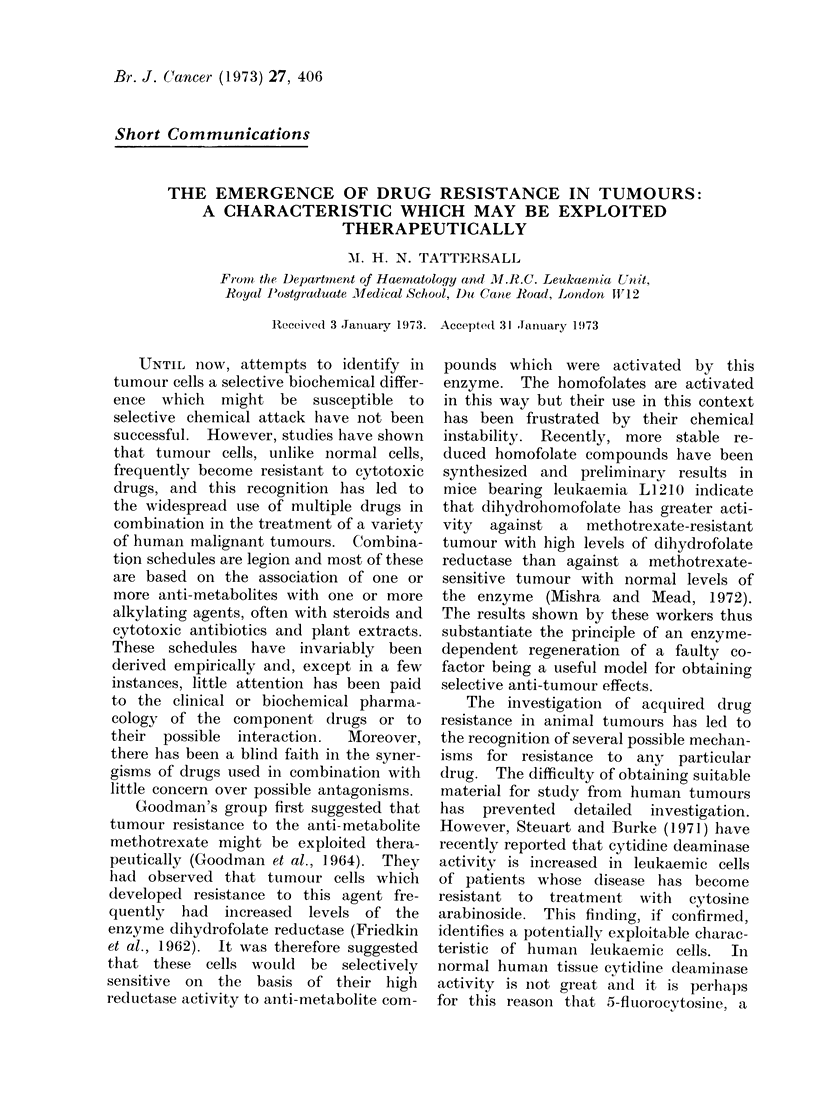

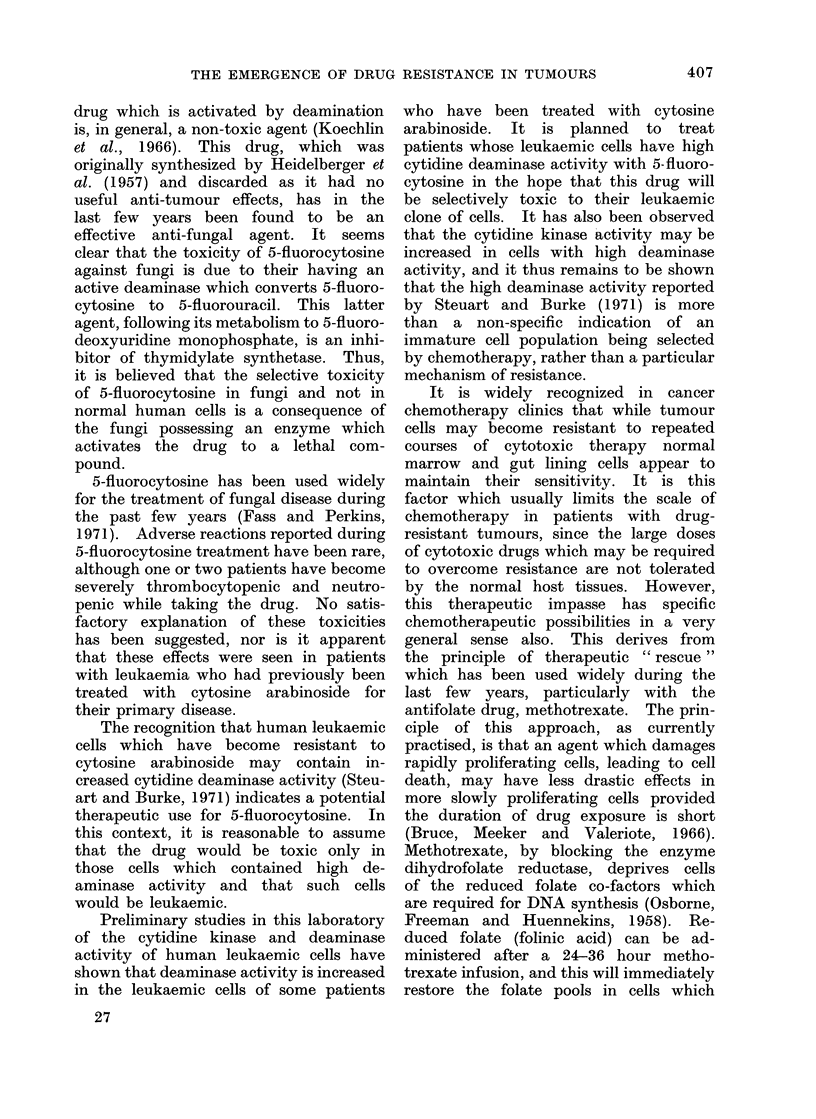

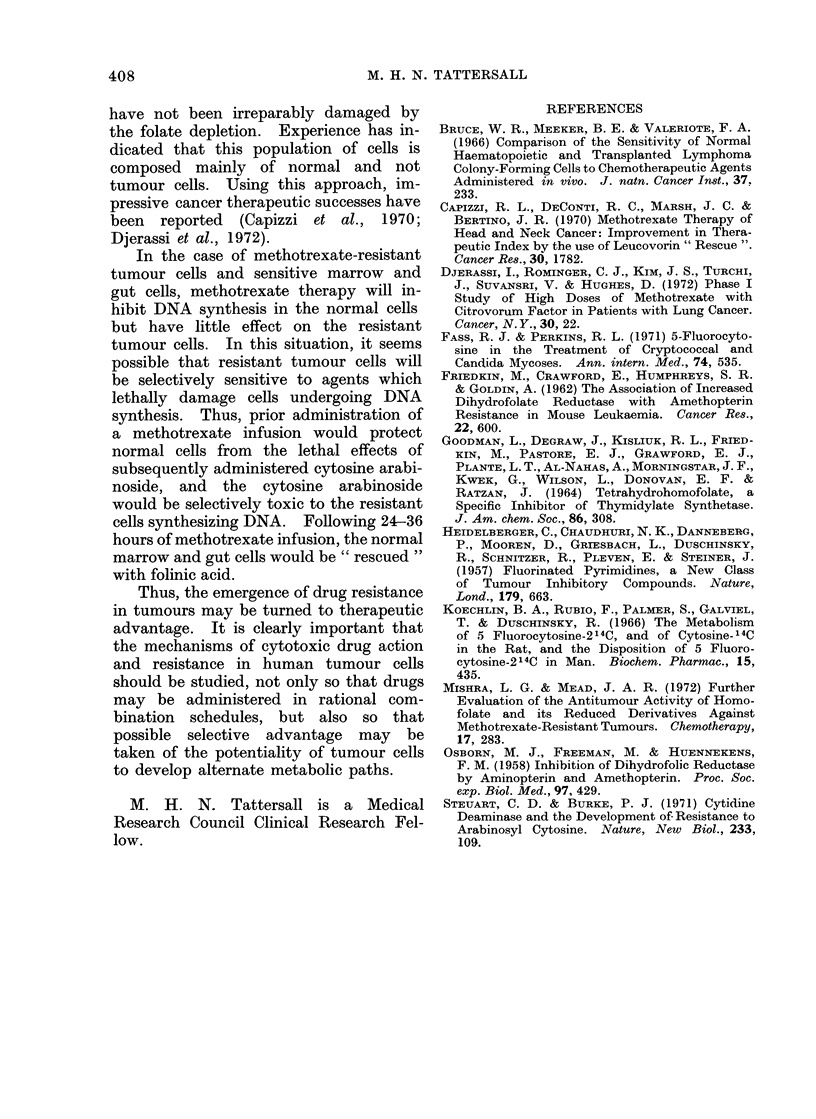


## References

[OCR_00260] Bruce W. R., Meeker B. E., Valeriote F. A. (1966). Comparison of the sensitivity of normal hematopoietic and transplanted lymphoma colony-forming cells to chemotherapeutic agents administered in vivo.. J Natl Cancer Inst.

[OCR_00268] Capizzi R. L., DeConti R. C., Marsh J. C., Bertino J. R. (1970). Methotrexate therapy of head and neck cancer: improvement in therapeutic index by the use of leucovorin "rescue".. Cancer Res.

[OCR_00275] Djerassi I., Rominger C. J., Kim J. S., Turchi J., Suvansri U., Hughes D. (1972). Phase I study of high doses of methotrexate with citrovorum factor in patients with lung cancer.. Cancer.

[OCR_00287] FRIEDKIN M., CRAWFORD E., HUMPHREYS S. R., GOLDIN A. (1962). The association of increased dihydrofolate reductase with amethopterin resistance in mouse leukemia.. Cancer Res.

[OCR_00282] Fass R. J., Perkins R. L. (1971). 5-fluorocytosine in the treatment of cryptococcal and candida mycoses.. Ann Intern Med.

[OCR_00303] HEIDELBERGER C., CHAUDHURI N. K., DANNEBERG P., MOOREN D., GRIESBACH L., DUSCHINSKY R., SCHNITZER R. J., PLEVEN E., SCHEINER J. (1957). Fluorinated pyrimidines, a new class of tumour-inhibitory compounds.. Nature.

[OCR_00311] Koechlin B. A., Rubio F., Palmer S., Gabriel T., Duschinsky R. (1966). The metabolism of 5-fluorocytosine-2-14-C and of cytosine-14-C in the rat and the disposition of 5-fluorocytosine-2-14-C in man.. Biochem Pharmacol.

[OCR_00319] Mishra L. C., Mead J. A. (1972). Further evaluation of the antitumor activity of homofolate and its reduced derivatives against methotrexate-insensitive tumors.. Chemotherapy.

[OCR_00326] OSBORN M. J., FREEMAN M., HUENNEKENS F. M. (1958). Inhibition of dihydrofolic reductase by aminopterin and amethopterin.. Proc Soc Exp Biol Med.

[OCR_00332] Steuart C. D., Burke P. J. (1971). Cytidine deaminase and the development of resistance to arabinosyl cytosine.. Nat New Biol.

